# Primary Extra Nodal Diffuse Large B-Cell Lymphoma of the Maxillary Sinus with Symptoms of Acute Pulpitis

**DOI:** 10.1155/2022/8875832

**Published:** 2022-04-07

**Authors:** Jian-tong Cui, Shao-qing Zhang, Hui-xia He

**Affiliations:** Department of Stomatology, The First Medical Center, Chinese PLA General Hospital, Beijing, 100853, China

## Abstract

Diffuse large B-cell lymphoma not otherwise specified (DLBCL-NOS) is a subtype of large B-cell non-Hodgkin lymphoma with various clinical and pathological manifestations. DLBCL-NOS which primarily arises from maxillary sinus is rare and hard to diagnose due to unique anatomy. Here, we present a case of DLBCL-NOS that developed in the left maxillary sinus of a 72-year-old male, who presented with severe toothache that resembled acute pulpitis. The lesion was diagnosed and treated based on radiographs, histological, immunohistological examinations, and PET-CT analysis. Despite its rare incidence, DLBCL-NOS should still be included in differential diagnoses to rule out malignancy in cases of endodontic disease.

## 1. Introduction

Diffuse large B-cell Lymphoma (DLBCL) is a group of malignant tumors with great heterogeneity in clinical manifestations, histological morphology, and prognosis. It is the most common type of non-Hodgkin lymphomas (NHL) which accounts for 30-40% of newly reported cases and mainly occurs in elderly men [1, 2]. Similar to NHL, DLBCL is divided into nodal DLBCL and extranodal DLBCL based on primary sites of origin [3]. Nodal DLBCL manifests as painless lymphadenopathy with symptoms of local compression or obstruction in early stage, while extranodal DLBCL has various clinical presentations depending on the extent of local invasion. DLBCL may occur at multiple locations; among which, the gastrointestinal tract is the most commonly affected site, being followed by the head and neck region [2]. Thus, patients with DLBCL have complex clinical manifestations (systemic symptoms involving stomach, lung, skin; B symptoms like fever, weight loss, etc.), histological morphology, and prognosis.

While previous literature has reported cases that occurred at the palate, alveolar ridge, and gingiva, DLBCL at the maxillary sinus has rarely been reported [4–7]. Early diagnosis of DLBCL at maxillary sinus is challenging. Due to its specific anatomy and large air space, a patient can be asymptomatic until the tumor perforates one of the walls. On the other hand, early symptoms resemble those of endodontic diseases, such as spontaneous pain, soft tissue swelling, sinus tract, hypnalgia, and sensitivity to percussion. The lack of unique symptoms and subtle clinical manifestations may obscure proper diagnosis and delay treatment. Due to the disease's aggressive and rapid progression, early diagnosis and treatment would be tremendously beneficial to DLBCL patients. Therefore, dentists should develop a deep understanding of DLBCL and conduct comprehensive examinations for patients with similar symptoms for differential diagnoses and treatments accordingly [2]. Here, we present an unusual case of DLBCL in a 72-year-old male that developed in left maxillary sinus, who initially presented with severe toothache and symptoms similar to those of acute pulpitis.

## 2. Case Report

A 72-year-old man presented to the Department of Stomatology at the Chinese PLA General Hospital with severe intermittent, spontaneous pain at night in the left maxillary posterior region for a week. Three days before his visit, the patient was diagnosed with apical periodontitis at maxillary left first premolar #12 (Universal Numbering System) and underwent pulpectomy in a local dental clinic. Since then, he has been taking analgesics and nonsteroidal anti-inflammatory medications, but severe spontaneous pain and hypnalgia were persistent.

**Figure 1:**
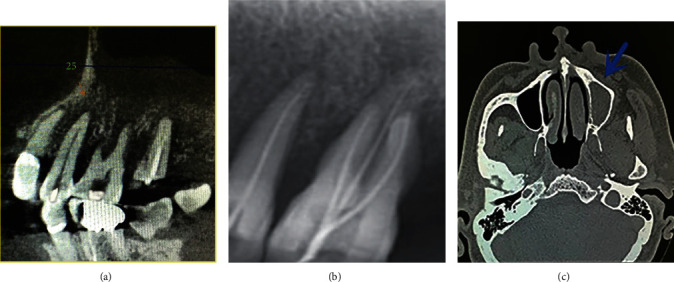
CBCT images showed multiple low-attenuating entities apical to premolars and the first molar. An extensive soft tissue mass was identified in the left maxillary sinus which extended through the medial maxillary sinus wall (a). Dental X-ray image showed appropriate root canal therapy (b). Transverse CT image showed maxillary sinus full of substantial objects which destroyed multiple walls (c).

No lymphadenopathy and swelling was noticed extraorally, yet intraorally poorly maintained dentition, and generalized edematous and erythematous gingiva were seen. There was extensive coronal structural loss for left maxillary second molar #15, which was endodontically treated 10 years ago, with fracture surface 2-3 mm under gingiva. The inflamed gingival margin was also observed buccally, and probing depth was 5-6 mm. Maxillary left first molar #14 had an exposed pulp cavity with mild mobility and a 5 mm periodontal pocket. The first and second left maxillary premolars, #12-#13, responded positively to percussion, and EPT test suggested necrotic pulps for #12-#14. A cone-beam computed tomography (CBCT) scan was performed instead of an initial Panoramic X-ray (PAN) because of its three-dimensional accuracy and the patient's preference for immediate appointment availability. Multiple well-defined hypo-attenuating entities were seen apical to #12-13 and #15 (Figure 1). Another was seen in the root canal of the residual root of #15, while an extensive soft tissue mass in the left maxillary sinus extending from medial maxillary sinus wall to palate at the left premolar and molar region was identified (Figure 1). As all symptoms suggested the diagnosis of pulpitis of #14 and the diagnosis of periapical periodontitis of teeth #12-13 and #15, root canal therapy was conducted for #12-#14, and residual #15 was removed (Figure 1). Computed tomography (CT) scan was performed, and sections were taken in the axial, coronal, and sagittal planes. Axial section revealed a large soft tissue density lesion almost full of the maxillary sinus, with destruction of multiple walls. It extends medially into the lateral wall of the nasal septum and right nasal cavity involving the inferior and middle turbinates and inferiorly causing destruction of the hard palate and alveolar processes of the left maxilla in the molar region (Figure 1). Left maxillary sinusitis was diagnosed, and the patient was given Ornidazole and Ceftriazone sodium for treatment. The patient reported slight alleviation of pain after the treatment. Yet, 14 days after the first visit, the patient presented to the department again with the chief complaint of recurrent pain at night in the upper molars region and facial paresthesia for 3 days.

**Figure 2:**
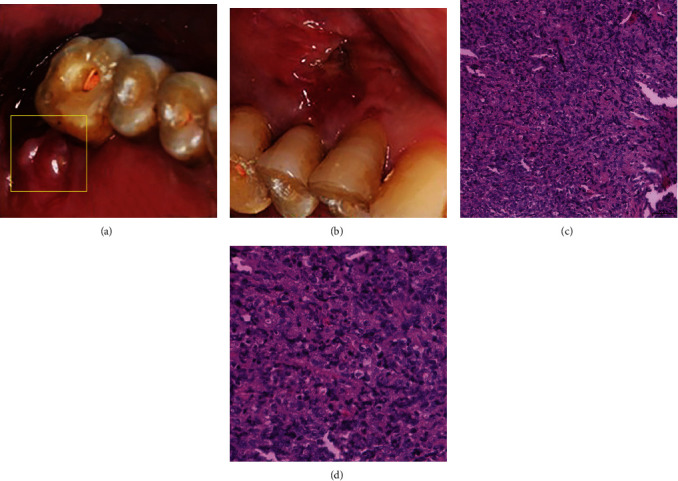
Two weeks after the first visit, intraoral pictures showed the poorly healed extraction socket of #15 with surrounding, localized, conglobate nontender edema (a) and a soft, well-defined, crater-like erythematous ulceration on the buccal gingiva of #13 and #14 (b). Histologic photomicrographs revealed a diffusely infiltrating population of neoplastic lymphoid cells within a fibrotic stroma with vascular channels (c, 200x), and the majority of lymphocytes were large cells (d, 400x).

**Figure 3:**
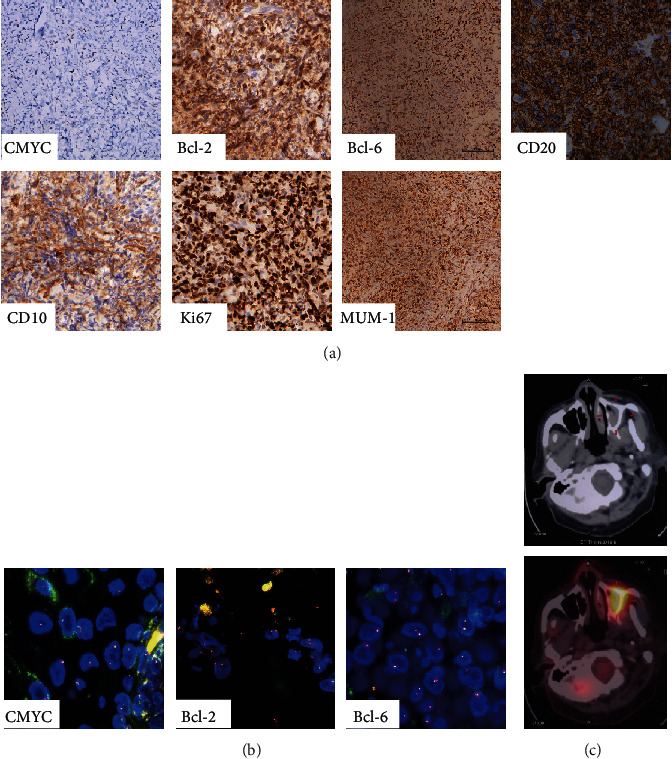
Immunohistochemical analysis showed that CD20, Bcl-2, Bcl-6, and MUM-1 were positively expressed. Ki-67 demonstrated a high (95%) proliferation rate, whereas CD10 and CMYC were negatively expressed (a). Fluorescence in situ hybridization (FISH) is negative for CMYC, Bcl-2, and Bcl-6 proto-oncogene rearrangements (b). Transverse PET-CT images showed about 2.8 × 3.3 cm hypermetabolic mass in the left maxillary sinus (c).

Intraorally, the extraction socket of #15 was poorly healed with localized surrounding nontender edema. A soft, well-defined, crater-like erythematous ulceration was also noticed on the buccal gingiva of #13 and #14 (Figure 2). Therefore, NHL, squamous cell carcinoma, sarcoma, plasmacytoma, and malignant melanoma were considered as differential diagnoses. Incisional biopsy for perilesional samples at the margin of the soft tissue mass at #15 revealed diffuse infiltration of neoplastic lymphoid cells within a fibrotic stroma with vascular channels (Figure 2). The majority of lymphocytes were large cells while a few were small ones manifesting as vesicular nuclei with prominent nucleoli. There were a few scattered foamy macrophages among those lymphocytes. Histology suggested the diagnosis of B-cell lymphoma. Positive expressions of CD20, Bcl-2, Bcl-6, and MUM-1 were observed in immunohistochemistry (IHC). Moreover, there was a strong expression of Ki67 (95%), whereas CD10 and CMYC were negatively expressed. Furthermore, CMYC, Bcl-2, and Bcl-6 proto-oncogene rearrangements diagnosed by fluorescence in situ hybridization (FISH) were negative (Figure 3). All of the evidence supported the diagnosis of DLBCL-NOS, cell of origin-post germinal center type, and nondouble expressor. Positron Emission Tomography-Computed Tomography (PET-CT) scan further suggested a hypermetabolic mass of about 2.8 × 3.3 cm^2^ in the left maxillary sinus, indicating the origin of the lymphoma (Figure 3).

**Figure 4:**
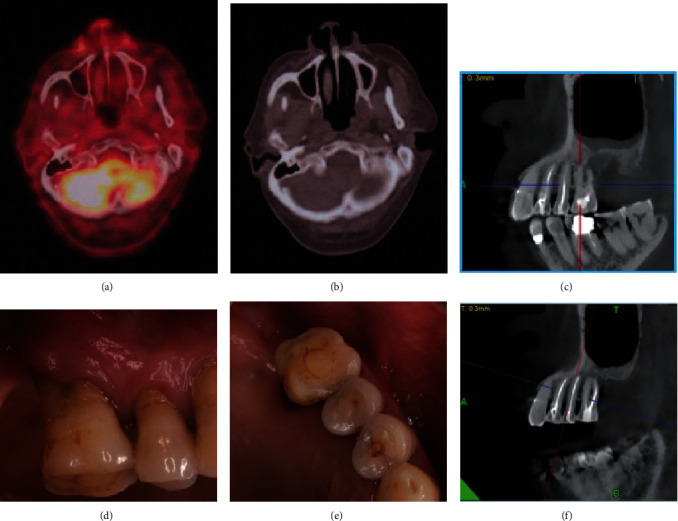
One month after the end of treatment, no distinct radioactive uptake in the left maxillary sinus was shown in transverse PET-CT images (a, b). CBCT image showed reduced substantial objects in the left maxillary sinus (c). Five years later, intraoral pictures showed healthy gingiva (d, e). CBCT showed that no substantial objects could be found in the left maxillary sinus (f).

The patient was referred to the Department of Oncology and received 6 cycles of standard chemotherapy regimen (R-CHOP, rituximab, cyclophosphamide, doxorubicin, vincristine, and prednisone). Rituximab 375 mg/m^2^ dl (day 1) (the first two-cycle), cyclophosphamide 750 mg/m^2^ dl, doxorubicin 50 mg/m^2^ dl, vincristine 2 mg/m^2^ dl, and prednisone 100 mg (days 1-5) were prescribed. Chemotherapy was administered in 3-week cycles, and the patient showed well tolerance to the treatment. The patient's symptoms were eliminated, and no intraoral swelling was identified at the end of the treatment. Imaging analysis of a follow-up PET-CT scan 1 month after the last chemotherapy session showed a negative result for isotope uptake at the left maxillary posterior region, and the CBCT image showed that substantial object in left maxillary sinus was reduced (Figure 4). The patient was followed up for five-year postchemotherapy, and no recurrence was seen to date (Figure 4).

## 3. Discussion

Identifying whether the source of toothache is of odontogenic origin (pulpitis, periapical periodontitis, dentin exposure, cracked tooth, etc.) or nonodontogenic origin (myofascial or neuropathic pain, malignancy, maxillary sinusitis, etc.) is critical yet sometimes challenging. Specifically, the similar symptoms of NHL in the maxillofacial region and those of odontogenic origin may potentially lead to misdiagnoses. The occurrence of DLBCL-NOS in the maxillary sinus, which is often asymptomatic or clinically similar to inflammatory disease, is extremely rare and difficult to be noticed due to its anatomic location and considerable size [3, 8, 9]. In our case, the patient presented with severe spontaneous pain and hypnalgia, while #12-#15 showed obvious tenderness and pulpal necrosis. The CBCT images ruled out the possibility of root fracture and showed diffuse periapical rarefaction associated with the molars and premolars in the left maxillary posterior area. Given that periapical endodontic disease could damage the wall of the maxillary sinus and no systemic symptoms of B-cell lymphoma including fever, weight loss, night sweats, and paresthesia were observed, we extracted the residual root of #15 and conducted endodontic therapy for the symptomatic teeth as usual rather than performing a biopsy.

Yet, the persistent symptoms (pain, unhealed extraction socket) raised our suspicion of pathologies of nonodontogenic sources in the maxillary sinus and led to appropriate workup. It is worthy of the clinician's attention that although periapical lesions are the most commonly encountered radiolucency in the jaws, about 0.65% to 4.22% of clinical cases were misdiagnosed as endodontic periapical pathosis before histopathological diagnoses were made [10]. In this case, further morphological examination showed that large B lymphoma cells were arranged in a spreading pattern with large nuclear and partially or completely covered normal lymph nodes or extranode structures. Histopathology result is the gold standard, reaching a definitive diagnosis of DLBCL rather than squamous cell carcinoma, sarcoma, plasmacytoma, or malignant melanoma [7]. Various morphologic variants of DLBCL-NOS have been described with centroblastic, immunoblastic, and anaplastic variants being the most common types [2]. A significant proportion of cases defy assignment into any specific subgroup. Combining with immunohistochemistry, diagnosis and histological grading of lymphoma could be established more accurately [2]. The expressions of CD10, Bcl-6, and MUM-1 are suggested to distinguish between germinal center B-cell-like (GCB) lymphoma and nongerminal center B-cell like (non-GCB). The positive expression of CD10 or negative expression of CD10 and MUM-1 with Bcl-6 positive expression should lead to identification of GCB tumor which implies a relatively better prognosis. The negative expression of CD10 and positive expression of MUM1 with positive or negative Bcl-6 indicate non-GCB tumor, which is often associated with a relatively poor prognosis [2, 7, 11]. Ki67 is a proliferative marker of neoplastic cells; therefore, the stronger expression of Ki67 is, the worse the prognosis will be. In our case, neoplastic cells were strongly positive for Ki67 (95%), MUM-1, Bcl-6, and Bcl-2 but negative for CD10. Both the histological examination and immunohistochemical analysis suggested the diagnosis of DLBCL NOS and cell of origin-post germinal center type, implying the aggressive characteristics and potentially poor prognosis in this patient if no timely diagnosis and treatment. It is worth noting that patients with a dual rearrangement of MYC and/or BCL2 and/or BCL6 “double-hit” lymphoma have been recognized to have a poor prognosis. In conjunction with FISH and PET-CT diagnosis, all diagnoses proved that it was DLBCL-NOS, cell of origin-post germinal center type, and nondouble expressor, which originated from the maxillary sinus that destroyed the bone plate of the maxillary sinus and the root of the teeth in the left maxillary region, causing pain similar to that of acute pulpitis.

DLBCL-NOS in the oral and maxillofacial regions may have very diverse manifestations including but not limited to local bone invasion, painless soft tissue swelling or ulceration, and toothache similar to that of periapical endodontic disease or pulpitis. Several articles have described delayed definitive diagnosis and relatively poor prognosis due to the atypical manifestation of maxillofacial lymphoma as toothache [4, 6, 12]. Fatahzadeh [4] reported a case of diffuse large B-cell lymphoma of mandible masquerading as toothache, in which etiology was not identified until the clinicopathologic examination was performed. Thus, the importance of proper differential diagnosis for early diagnosis could not be undermined. Comprehensive inspection including clinical evaluation, radiographic examination, histological assessment, and immunophenotypic analysis is necessary for diagnosis and treatment.

To date, the mainstay of the treatment regimen for DLBCL-NOS consists primarily of chemotherapy with the occasional use of radiotherapy. Cyclophosphamide, doxorubicin, vincristine, and prednisone (CHOP) are part of a widely used regimen. Anti-CD20 medication (rituximab) improves complete remission rates in NHL patients while it seems to prolong overall survival, although about 30%-40% of DLBCL patients with CD20 positive are refractory to R-CHOP therapy [2]. For these patients, other treatments such as autologous stem cell transplantation, R-EPOCH (rituximab, etoposide, prednisone, vincristine, cyclophosphamide, and doxorubicin), targeted theories, and novel approaches (chimeric antigen receptor- (CAR-) T cell therapy) could be considered [13]. Recently, many targeted drugs that inhibit signaling pathways in DLBCL cells have entered clinical trials, and many therapeutic agents are available on market. Treatment strategies are also tailored according to DLBCL-NOS patients' risk profiles according to age and age-adapted International Prognostic Index (IPI) [14]. Considering that 6-8 cycles of combined chemotherapy with R-CHOP-21 is the most widely used regimen for patients from 60 to 80 years of age, rituximab was added to the CHOP regimen for the patient in our case, though there is limited experience of the use of anti-CD20 medication during maintenance or consolidation for NHL initial presentation. The average time, reported in previous literature, to arrive at a definitive diagnosis for lymphoma which manifested as a toothache was approximately 5 months [5, 15, 16], while that of our case was about 1 month. With comprehensive inspection and prompt treatment, the patient's symptoms resolved, and no recurrence was seen in the following 5 years.

In summary, we described an unusual case of DLBCL-NOS which initially occurred in the left maxillary sinus and was associated with localized endodontic and periapical disease in a 72-year-old male. This report stresses that dentists should be alert to the possibility of malignancy in the maxillary sinus, especially when the patient presents with persistent maxillofacial pain after involved teeth were managed. Early radiological and histological examinations are encouraged to achieve an accurate diagnosis, precise treatments, and a favorable prognosis.
